# Clinic Versus the Operating Room: Determining the Optimal Setting for Dilation and Curettage for Management of First-Trimester Pregnancy Failure

**DOI:** 10.7759/cureus.56490

**Published:** 2024-03-19

**Authors:** Hilary Novatt, Kari Rockhill, Kori Baker, Elaine Stickrath, Meredith Alston, Stefka Fabbri

**Affiliations:** 1 Obstetrics and Gynecology, University of Colorado School of Medicine, Aurora, USA; 2 Epidemiology and Public Health, Rocky Mountain Poison & Drug Safety, Denver, USA; 3 Obstetrics and Gynecology, UCHealth Women’s Care Clinic, Steamboat Springs, USA; 4 Obstetrics and Gynecology, Intermountain Health Saint Joseph Hospital, Denver, USA; 5 Obstetrics and Gynaecology, Denver Health, Denver, USA

**Keywords:** outpatient setting, surgical and clinical management, miscarriage, first trimester pregnancy failure, dilation and curettage

## Abstract

Introduction

There is no clear guidance for the optimal setting for dilation and curettage (D&C) for the management of first-trimester pregnancy failure. Identifying patients at risk of clinically significant blood loss at the time of D&C may inform a provider's decision regarding the setting for the procedure. We aimed to identify risk factors predictive for blood loss of 200mL or greater at the time of D&C.

Methods

This is a retrospective cohort study of patients diagnosed with first-trimester pregnancy failure at gestational age less than 11 weeks who underwent surgical management with D&C at a single safety net academic institution between 4/2016 and 4/2021. Patient characteristics and procedural outcomes were abstracted. Women with less than 200mL versus greater than or equal to 200mL blood loss were compared using descriptive statistics, chi-square for categorical variables, and Satterthwaite t-tests for continuous variables.

Results

A total of 350 patients were identified; 233 met inclusion criteria, and 228 had non-missing outcome data. Mean gestational age was 55 days (SD 9.4). Thirty-one percent (n=70) had estimated blood loss (EBL) ≥200mL. Younger patients (mean 28.7 years vs. 30.9, p=0.038), Latina patients (67.1% vs. 51.9%, p=0.006), patients with higher body mass index (BMI, mean 30.6 vs. 27.3 kg/m2, p=0.006), and patients with pregnancies at greater gestational age (59.5 days vs. 53.6 days, p<0.001) were more likely to have EBL ≥200mL. Additionally, patients with pregnancies dated by ultrasound (34.3% vs. 18.4%, p=0.007), those who underwent D&C in the operating room (81.4% vs. 48.7%, p<0.001), and those who underwent general anesthesia (81.4% vs. 44.3%, p<0.001) were more likely to have EBL ≥200mL.

Discussion

In this study, patients with EBL ≥200mL at the time of D&C differed significantly from those with EBL<200mL. This information can assist providers in planning the best setting for their patients' procedures.

## Introduction

Early pregnancy failure, also referred to as missed abortion or miscarriage, is common, occurring in 10% of all clinically recognized pregnancies [1,2]. There are several management options for early pregnancy failure, including expectant, medical, or surgical management. Surgical management, typically performed with suction curettage by manual vacuum aspiration (MVA) or electric vacuum aspiration (EVA), is recommended for hemorrhage, hemodynamic instability, or infection. It is also offered based on patient preference.

Despite the frequency of the procedure and its documented safety [3,4], there is no agreement on the definition of clinically significant bleeding at the time of dilation and curettage (D&C). The only studies examining risk factors for increased blood loss at the time of D&C for first-trimester pregnancy failure evaluated procedural factors, such as surgical technique or anesthetic use [3,5-10].

There is also a lack of information as to which patient characteristics are associated with increased blood loss at the time of uterine evacuation for first-trimester pregnancy failure. Some studies have examined the association of patient demographics with blood loss at the time of second-trimester pregnancy termination [11-18]. However, this is a different clinical scenario than first-trimester pregnancy failure. The authors were unable to identify any published studies examining patient characteristics associated with increased blood loss in the setting of D&C for first-trimester pregnancy failure (PubMed search 3/5/2021, search words "blood loss," "bleeding," or "hemorrhage" and "first-trimester pregnancy" or "miscarriage" and "abortion," "dilation and curettage," "manual vacuum aspiration," or "termination" and "clinic," "outpatient," or "operating room").

Identifying which patients with early pregnancy failure are at higher risk of clinically significant blood loss at the time of D&C is of great interest, as this information may help providers decide on the best setting in which to perform this procedure. MVA can be performed safely in the outpatient setting [19], which may be a preferred option overutilization of the operating room given the significant resource and cost savings [20]. However, patients expected to have an increased risk of bleeding may be better served in the operating room, as unexpected bleeding in the outpatient setting can be associated with increased morbidity related to emergent transfer to the operating room and delayed management of hemorrhage.

At our institution, we have historically offered surgical management of first-trimester pregnancy failure for patients less than 11 weeks gestation by crown-rump length (CRL), either in the outpatient setting or in the operating room, based on provider recommendation and patient preference without clear institutional guidance. For this study, the authors arbitrarily selected 200 mL as clinically significant blood loss for the procedure. Thus, the study aimed to identify risk factors associated with an estimated blood loss (EBL) of 200mL or greater at the time of D&C to manage first-trimester pregnancy failure.

## Materials and methods

We performed a retrospective cohort study at a single safety-net academic hospital between April 2016 and April 2021. Patients were included if they were 18 to 45 years of age, were diagnosed with first-trimester pregnancy failure at gestational age less than 11 weeks 0 days, and who elected or were recommended surgical management were included. Patients were excluded if they were incarcerated, had concerns about molar pregnancy, or were concerned about invasive placentation. Patients undergoing emergency D&C were also excluded.

Early pregnancy failure was diagnosed based on published clinical guidelines including: CRL of 7mm or greater and no cardiac activity; mean sac diameter (MSD) of 25mm or greater and no embryonic structures; absence of embryo with cardiac activity two weeks or more after a scan that showed a gestational sac (GS) without a yolk sac (YS); and absence of embryo with cardiac activity 11 days or more after a scan that showed a GS with a YS [2].

The primary outcome was EBL greater than or equal to 200mL. The cut-off of 200ml was arbitrarily chosen based on provider consensus and data that this degree of blood loss is generally higher than expected for a first-trimester procedure [12].

Patients who met inclusion criteria were identified through current procedural terminology (CPT) codes and a review of the electronic medical record (EMR). Demographic and obstetric characteristics, medical comorbidities, and treatment courses, such as failure of expectant and/or medical management, were abstracted from the medical record. In addition, procedure characteristics such as location, type of evacuation, type of anesthesia, use of uterotonics and/or tranexamic acid, and procedural complications, including emergency transfer to the operating room, were also abstracted through EMR reports and manual record review. 

Gestational age for each patient was collected and categorized based on ultrasound performed at the time of diagnosis of pregnancy failure into the following groups: pregnancies with an empty GS measuring less than 25mm by MSD, an empty GS greater than 25mm MSD, or a GS with a YS but absence of fetal pole. The length of time between the initial diagnosis of the pregnancy failure and uterine evacuation was collected. We also calculated an estimated duration of pregnancy failure before evacuation when possible, based on chart review (documented sure last menstrual period (LMP), positive pregnancy test, or prior ultrasound in the current pregnancy).

Demographic characteristics of patients with blood loss less than 200mL and greater than or equal to 200mL were compared using descriptive statistics. Univariate analysis was completed with ANOVA or Wilcoxon two-sample t-test for continuous variables and Chi-square test or Fisher's Exact Test for categorical variables based on data distributions. Statistical analysis was performed using SAS Version 9.4 (Cary, North Carolina). The study was reviewed and exempt from IRB review due to its designation as secondary research.

## Results

**Figure 1:**
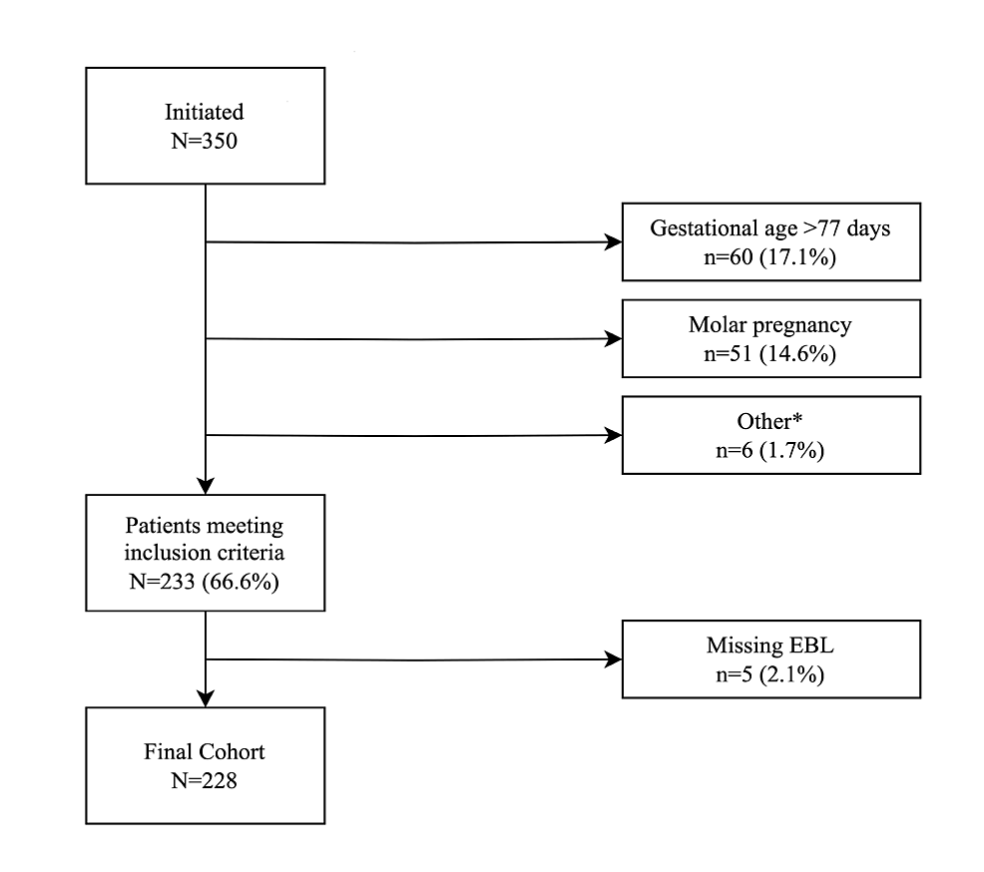
Cohort selection * Included patients who were incarcerated, had a known bleeding disorder, suspected invasive placentation, or who underwent emergent D&C. D&C: Dilation and Curettage; EBL: Estimated blood loss

A total of 350 patients were identified. Of those, 122 were excluded based on exclusion criteria. Cohort selection is presented in (Figure 1).

Excluded patients had no significant differences when comparing age, race, ethnicity, history of bleeding disorder, history of prior uterine surgery, body mass index (BMI, kg/m^2^), gravidity, or parity (data not shown).

Two hundred twenty-eight patients met the inclusion criteria. Demographic and procedural characteristics are summarized in (Table 1). The majority of the patients were Latina (n=129, 56.6%) and publicly insured (n=136, 59.6%), with a mean gestational age of 55.3 days (SD-9.4). In most cases (n=157, 68.9%), the pregnancy was dated by LMP. The median duration from diagnosis of pregnancy failure to the procedure was five days (IQR: 3-11). Thirty-nine patients (17.1%) had failed expectant management, and 24 patients (10.5%) had failed medical management before undergoing D&C. More patients underwent D&C in the operating room (N=134, 58.8%) than in the outpatient setting (N=94, 41.2%).

**Table 1:** Patient and procedural characteristics by blood loss at the time of dilation and curettage for first-trimester pregnancy failure.* *Data shown as N (%) unless otherwise indicated. SD: standard deviation; BMI: body mass index; IQR: interquartile range; MAC: monitored anesthesia care (intravenous sedation); EBL: estimated blood loss; LMP: last menstrual period

	Total Sample N=228	EBL <200mL N=158 (69.3%)	EBL ≥200ml N=70 (30.7%)	p-value
Age at Procedure, mean (SD)	30.2 (7.0)	30.9 (6.7)	28.7 (7.5)	0.038
Race				
White	149 (65.4%)	102 (64.6%)	47 (67.1%)	0.705
Black	33 (14.5%)	23 (14.6%)	10 (14.3%)	0.957
Other/Unknown	37 (16.2%)	26 (16.5%)	11 (15.7%)	0.568
Ethnicity				0.006
Latina	129 (56.6%)	82 (51.9%)	47 (67.1%)	
Non-Latina	97 (42.5%)	74 (46.8%)	23 (32.9%)	
Unknown	2 (0.9%)	2 (1.3%)	0 (0.0%)	
BMI (kg/m^2^), mean (SD)	28.3 (7.2)	27.3 (5.9)	30.6 (9.2)	0.006
Gravidity, median (IQR)	3 (2, 5)	3 (2, 5)	3 (2, 5)	0.338
Parity, median (IQR)	1 (0, 3)	1 (0, 2)	1 (0, 3)	0.791
Insurance Category				0.167
Medicaid	136 (59.6%)	87 (55.1%)	49 (70.0%)	
Private Insurance	40 (17.5%)	32 (20.3%)	8 (11.4%)	
Uninsured or Assistance Program	41 (18.0%)	30 (19.0%)	11 (15.7%)	
Other/Unknown	11 (4.8%)	9 (5.7%)	2 (2.9%)	
History of Hypertension	18 (7.9%)	13 (8.2%)	5 (7.1%)	0.779
History of Diabetes Mellitus	13 (5.7%)	10 (6.3%)	3 (4.3%)	0.759
History of Prior Uterine Surgery	81 (35.5%)	58 (36.7%)	23 (32.9%)	0.575
Multiple Gestation	9 (3.9%)	4 (2.5%)	5 (7.1%)	0.138
Gestational Age (days), Mean, (SD)	55.3 (9.4)	53.6 (8.9)	59.5 (9.5)	<0.001
Gestational Age Typing				0.007
LMP	157 (68.9%)	119 (75.3%)	38 (54.3%)	
Ultrasound	53 (23.2%)	29 (18.4%)	24 (34.3%)	
Unknown	18 (7.9%)	10 (6.3%)	8 (11.4%)	
Duration of Pregnancy Failure (days), mean (SD)	31.5 (20.5)	31.6 (20.0)	31.4 (22.0)	0.964
Duration from diagnosis of Failure to Procedure (days), median (IQR)	5 (3, 11)	5 (2, 10)	6 (3, 11)	0.477
Failure of Expectant Management	39 (17.1%)	26 (16.5%)	13 (18.6%)	0.696
Failure of Medical Management	24 (10.5%)	18 (11.4%)	6 (8.6%)	0.522
Location of Procedure				<0.001
Outpatient	94 (41.2%)	81 (51.3%)	13 (18.6%)	
Operating Room	134 (58.8%)	77 (48.7%)	57 (81.4%)	
Anesthetic				<0.001
General	127 (55.7%)	70 (44.3%)	57 (81.4%)	
Local Anesthetic	119 (52.2%)	95 (60.1%)	24 (34.3%)	
Oral Medications	84 (36.8%)	74 (46.8%)	10 (14.3%)	
MAC	20 (8.8%)	16 (10.1%)	4 (5.7%)	
Other	1 (0.4%)	1 (0.6%)	0 (0.0%)	

**Figure 2:**
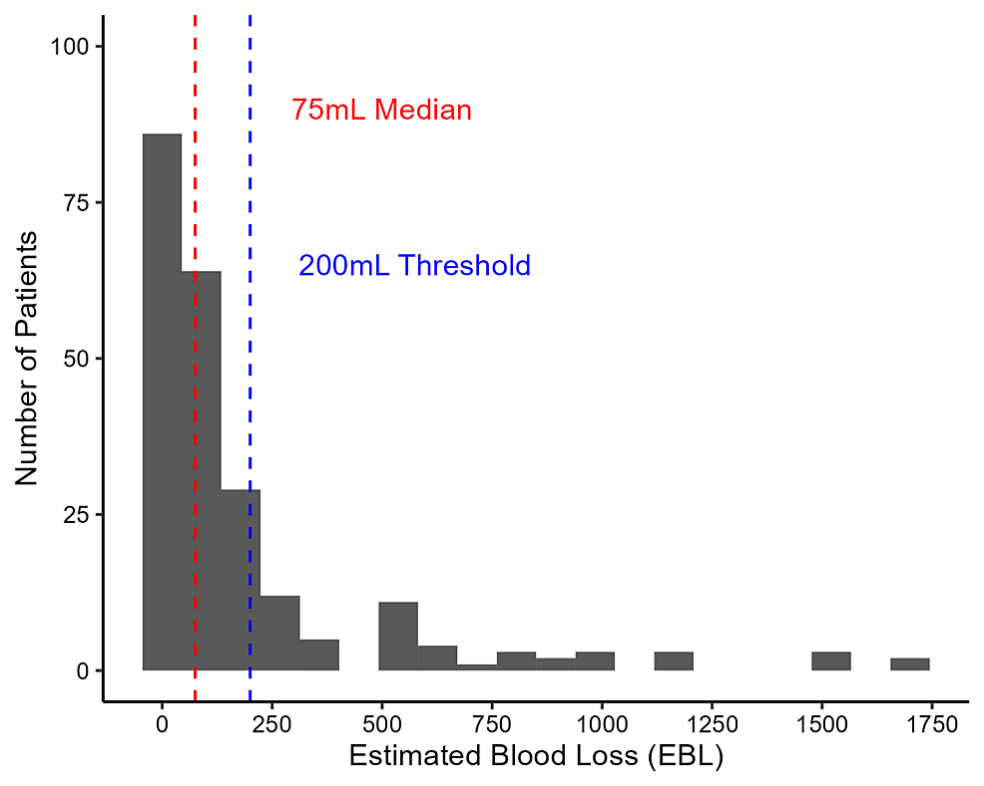
Blood loss distribution among patients undergoing dilation and curettage for early pregnancy failure The median estimated blood loss (EBL) for the entire cohort was 75mL. Seventy patients (30.7%) had EBL greater than or equal to 200mL, and 158 patients (69.3%) had EBL less than 200mL. Those with "minimal" blood loss are shown visually in the zero bin.

Overall, for the entire cohort, the median blood loss at the time of D&C was 75mL (IQR: 20-200mL). One hundred fifty-eight patients (69.3%) had blood loss less than 200mL, and 70 (30.7%) had blood loss greater than or equal to 200mL. Blood loss distribution is shown in (Figure 2). Thirty-seven patients (16.2%) had documented blood loss of "minimal" rather than a discrete number. These patients were considered to have blood loss less than 200mL. A total of 32 (14.0%) patients had blood loss of 500mL or more, and the majority of these procedures (31, 13.6%) were performed in the operating room.

Younger age (mean 28.7 years vs. 30.9, p=0.038), Latina ethnicity (67.1% vs. 51.9%, p=0.006), and higher BMI (mean 30.6 vs. 27.3 kg/m^2^, p=0.006) were significantly associated with EBL greater than or equal to 200mL compared to less than 200mL (Table 1). Greater gestational age (59.5 days vs. 53.6 days, p<0.001), pregnancies dated by ultrasound (34.3% vs. 18.4%, p=0.007), those who underwent D&C in the operating room (81.4% vs. 48.7%, p<0.001), and those who underwent general anesthesia (81.4% vs. 44.3%, p<0.001) were more likely to have EBL greater than or equal to 200mL (Table 1). Estimated/calculated duration of pregnancy failure, as well as duration from diagnosis of pregnancy failure to the procedure, were not found to be significantly associated with greater blood loss.

## Discussion

For patients undergoing D&C for first-trimester pregnancy failure, the median blood loss was 75mL. Younger age, Latina ethnicity, higher BMI, greater gestational age, ultrasound dating, procedure in the operating room, and general anesthesia were significantly associated with EBL greater than or equal to 200mL. The significant association of higher BMI, greater gestational age, and the use of general anesthesia with increased bleeding during first-trimester uterine evacuation is consistent with data involving second-trimester D&C as well as postpartum hemorrhage [9,12]. However, the latter can be related to provider selection bias, where patients perceived to be at higher risk for increased bleeding are selectively scheduled for the operating room.

There is no biological plausibility for Latina ethnicity being associated with increased bleeding at the time of the first-trimester D&C. This finding may be related to societal influences on healthcare outcomes. Factors such as socioeconomic status, access to healthcare services, insurance coverage, cultural beliefs and practices, and systemic inequalities may predispose certain populations to adverse health outcomes [21]. Additional factors, such as language barriers and mistrust in healthcare systems, also play a role [22]. Recognizing that income level, education, and occupation are among the various factors influencing access to healthcare resources and preventative measures, it must be acknowledged that lower socioeconomic status is often linked with limited access to quality healthcare services, including prenatal care [22,23]. Consequently, these underrepresented patients may have delayed diagnosis and management of pregnancy-related conditions that could lead to more complex presentations during medical procedures like D&C [24].

Among the strengths of this study is the novel investigation of multiple factors associated with an elevated risk of higher EBL in first trimester D&C. In addition, extensive manual review of the electronic medical record ensured minimal missing data. Only five patients (2%) had missing blood loss at the time of the procedure. The study has several notable limitations. It is possible that the assumption that minimal blood loss corresponded to EBL less than 200mL is untrue. The latter can lead to misclassification of patients and skew the results toward the null hypothesis. As noted previously, the study's retrospective nature and the lack of randomization can be a source of selection bias where patients perceived to be at higher risk for increased bleeding are preferentially scheduled for the operating room. Various factors may have influenced the decision for patients to undergo D&C in the operating room rather than in an outpatient setting. Individual patient concerns and preferences could have played a role in this decision. Within our cohort, we observed a higher likelihood of patients at an advanced gestational age undergoing the procedure in the OR. This tendency may be attributed to provider recommendations influenced by their bleeding risk assessment.

Additionally, it is important to consider potential confounding variables that may contribute to increased blood loss during D&C. For instance, patients dated by ultrasound were more likely to have a greater gestational age during the procedure (p<0.001). Thus, the increased blood loss may be associated more with the advanced gestational age than the mode of dating, as previous studies have indicated a correlation between greater gestational age and increased blood loss [12]. Moreover, using general anesthesia could introduce a confounding factor when assessing the impact of the OR setting on blood loss. General anesthesia has been associated with increased blood loss in various clinical scenarios, including procedures such as dilation and evacuation (D&E) for second-trimester pregnancy termination and cesarean section [9]. Future studies could explore these unconfounded relationships further.

As a single-site study with a relatively small sample size, the generalizability of our findings to other institutions may be limited. Additionally, blood loss is frequently reported as an estimate, and there is ample evidence showing blood loss estimation underreports actual blood loss [25,26]. The accuracy of this measure may be higher in the operating room compared to the outpatient setting, given providers may frequently rely on graduated canisters for blood loss estimation. Further, missing information, such as lack of early dating ultrasound or unsure last menstrual period in patients with irregular menses, made it difficult to determine gestational age and duration of pregnancy failure for some patients.

## Conclusions

This study is one of the initial investigations aimed at identifying risk factors associated with blood loss of 200mL or greater at the time of D&C for the management of first-trimester pregnancy failure. This retrospective cohort study revealed significant associations between greater blood loss and various patient characteristics, including demographic factors and health-related attributes. Identifying the modifiable and non-modifiable factors may provide insights for clinical decision-making regarding the optimal setting for the first-trimester D&C procedure. Additional studies are warranted to make robust recommendations and to validate these associations.
